# Compressive Stress–Strain Relationship of Recycled Coarse Aggregate Concrete After Sulfate Corrosion and High Temperature

**DOI:** 10.3390/ma19030477

**Published:** 2026-01-24

**Authors:** Ziliang Cai, Jin Wu, Xing Zhao, Xiaoxia Lu, Lifang Zhang, Yiyuan Wang, Haoxiang Luan

**Affiliations:** 1 College of Civil Aviation, Nanjing University of Aeronautics and Astronautics, No. 29, Yudao St., Nanjing 211106, China; 2 Eastern Airport Co., Ltd., Nanjing 211100, China; 3 College of Architectural Engineering, Jiangsu Open University, Nanjing 210036, China

**Keywords:** recycled aggregate concrete, dry–wet cycle, sulfate corrosion, high temperature, stress–strain curves

## Abstract

Structures or chemical engineering facilities can be subjected to the combined effects of sulfate corrosion and high temperature in the event of fire. This paper presents experimental results on compressive stress–strain relationships of recycled coarse aggregate concrete (RAC) and normal aggregate concrete (NAC) after dry–wet cycles of sulfate corrosion and high-temperature exposure. First, RAC and NAC specimens were subjected to 0, 20, 40, 60, 80, 100, and 120 dry–wet cycles of sulfate corrosion, respectively. Then, RAC and NAC specimens were subjected to 0 °C, 200 °C, 400 °C, 600 °C, and 800 °C temperature exposures, respectively. At last, RAC and NAC specimens were loaded by uniaxial compressive test. The test results show that the shapes of the stress–strain curves of RAC and NAC specimens after the 200 °C exposure and dry–wet cycles of sulfate corrosion were basically the same as those at room temperature. When the temperature was in the range of 200–400 °C, the elastic modulus and peak stress of RAC decreased with the number of dry–wet cycles of sulfate corrosion, while the corresponding peak strain gradually increased. When the temperature was lower than 400 °C, the number of dry–wet cycles of sulfate corrosion had a greater impact on the peak strain of RAC, while the temperature had a greater impact on the peak strain of RAC when the temperature exceeded 400 °C. After the temperature exceeded 400 °C, the elastic part in the ascending section of the stress–strain curve of RAC gradually shortened, and the peak point of the curve also shifted significantly to the lower right. The increase in peak strain of the RAC was larger than that of NAC. Based on the test results, a compressive stress–strain relationship model of RAC after sulfate corrosion and high temperature is established.

## 1. Introduction

In the process of urbanization, a large number of old buildings and structures are demolished, resulting in a sharp increase in the production of waste concrete [1,2,3,4]. Construction and demolition waste (CDW) can account for 30–40% of the total urban waste in China, with an annual output of 40–50 million tons [2,5]. Recycled aggregate concrete (RAC) refers to the concrete prepared by crushing, cleaning, and grading the waste concrete blocks partially or completely replacing natural aggregates. The application of RAC can not only maximize the conservation of natural aggregates to protect the ecological environment of aggregate production areas but also solve problems such as land occupation and environmental pollution caused by treatment of waste concrete.

Due to the wide application of concrete materials in high-temperature resistance and fire prevention fields, numerous scholars have conducted extensive research on its relevant properties after high-temperature exposure, especially the mechanical properties after high temperature [6,7,8,9,10,11,12]. It has been shown that the strength of RAC decreases after high-temperature exposure. Type of recycled aggregate [6,8,10,11], replacement level [8,9] and strength at ambient temperature [6,11] can affect the mechanical properties of RAC after high-temperature exposure. The overall shape of the stress–strain curves of RAC is basically similar to that of natural aggregate concrete (NAC). The stress–strain curves of RAC tend to become flatter as the temperature gradually increases. By analyzing the influence of various test variables on the mechanical properties of RAC after high-temperature exposure, an evaluation formula for the axial compressive strength of RAC after high-temperature exposure can be proposed [10,13,14,15,16].

Sulfate attack is another critical threat for concrete structures in marine and coastal areas, on roads with deicing salts, in saline-alkaline lands, and in industrial salt environments, as aggressive ions (e.g., SO_4_^2−^ in magnesium sulfate) react with cement hydration products to form expansive phases (ettringite, gypsum) or weak gels (M-S-H), leading to cracking and strength degradation [17,18,19,20,21,22,23]. RAC, with its higher porosity and porous interfacial transition zones (ITZs) between RCA and new mortar, is more susceptible to sulfate penetration than NAC [20,21,24,25]. However, fewer studies have reported on the recycled aggregate concrete after combination effect of high temperature and sulfate corrosion, and no compressive constitutive model of recycled aggregate concrete under the combined action of the two factors has been available.

In this paper, the compressive performance, failure modes and damage evolution laws of recycled aggregate concrete after both high temperature and dry–wet cycles of sulfate corrosion exposure are explored. A compressive stress–strain model of RAC after high temperature and sulfate corrosion is established. This study provides substantial support for predicting the performance of recycled concrete under the combined action of corrosion and elevated temperatures, as well as for the fire-protection design of offshore engineering structures or chemical engineering facilities.

## 2. Experimental Program

### 2.1. Experimental Materials

In this test, the “Conch brand” P.O 42.5 ordinary Portland cement (Wuhu, China) was used, and its performance indicators are shown in Table 1.

**Figure 1:**
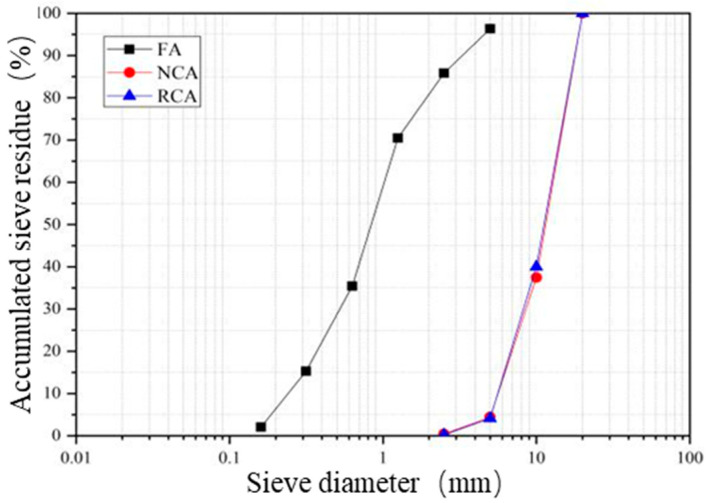
Aggregate Gradation Curves.

In this test, natural river sand was used as fine aggregate (FA) in the concrete. According to the test methods in the “Standard for Quality and Inspection Methods of Sand and Stone for Ordinary Concrete” (JGJ52-2006) [26], the natural river sand belongs to Grading Zone II. The grading distribution curve is shown in Figure 1. It has an apparent density of 2536 kg/m^3^, a fineness modulus of 2.76, and a clay content of 1.3% (<2.0%), which is classified as medium sand. In this test, the coarse aggregates are RCA (Recycled Coarse Aggregate) and NCA (Natural Coarse Aggregate). The maximum particle size of both coarse aggregates is 20 mm, and their grading curves are shown in Figure 1.

The properties of the NCA and RCA used in this experiment are shown in Table 2.

After trial mixing, the concrete mix proportions are shown in Table 3.

### 2.2. Specimen Design

Studies [27,28] have shown that the specimens for quasi-static tests should preferably have a length-to-diameter ratio of 2. Molds with a height of approximately 150 mm and a diameter of 70 mm were made using PVC pipes. It should be noted that the specimen size of φ70×140mm is smaller than the standard size of diameter of 150 mm and height of 300 mm because of the height limitation of the high-temperature furnace used in the test. The specimen ID adopts the form of R-X-Y or N-X-Y, where R and N represent recycled concrete specimens and normal concrete specimens, respectively. X represents corrosion cycles of sulfate corrosion, and Y represents the temperature value of high-temperature exposure. For example, R-10-200 indicates a RAC specimen that had undergone 10 dry–wet cycles of sulfate corrosions and 200 °C high-temperature exposure. The details are shown in Table 4. The NAC specimens were tested only up to the condition of 80 dry–wet cycles of sulfate corrosion and 400 °C high-temperature exposure (N-80-400), while RAC specimens covered a broader scope (0–120 corrosion cycles and 20–800 °C) based on the core research focus on RAC’s performance under the combined effects of sulfate corrosion and high temperature

The recycled coarse aggregate (RCA) was pre-soaked with tap water for about 30 min and drained for 20 min to reach a saturated-surface-dry condition before mixing. All the concrete specimens were cast in accordance with the “Code for Mix Proportion Design of Ordinary Concrete” (JGJ55-2011) [29] and the “Standard for Test Methods of Mechanical Properties of Ordinary Concrete” (GB/T 50081-2016) [28].

## 3. Test Program

### 3.1. Sulfate Corrosion

**Figure 2:**
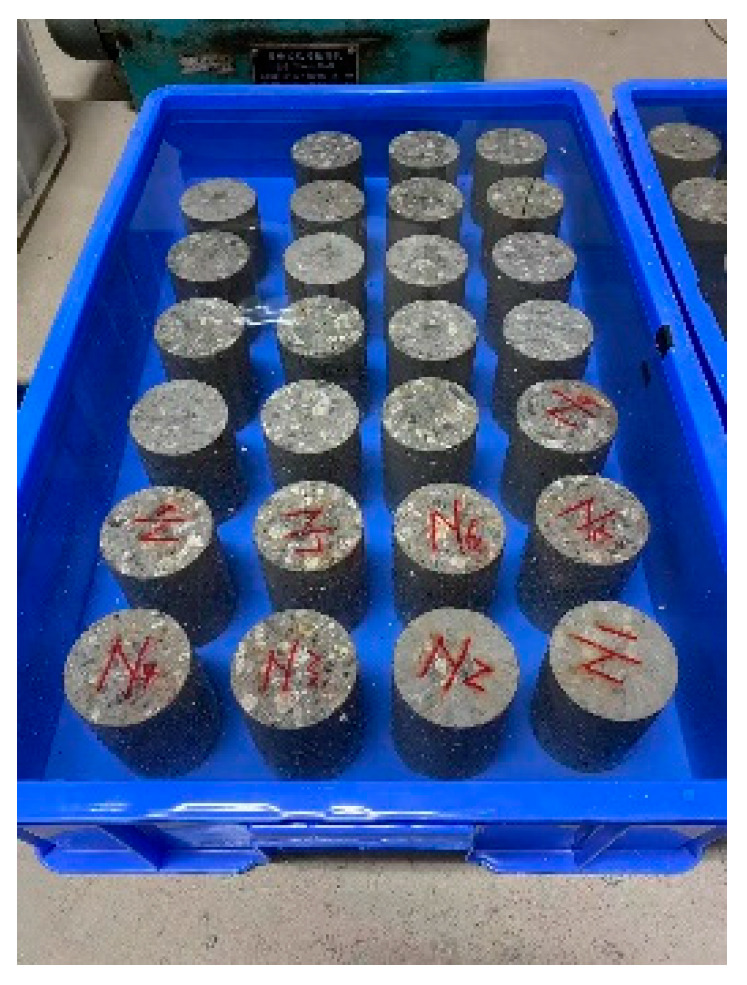
Sulfate Solution Soaking.

**Figure 3:**
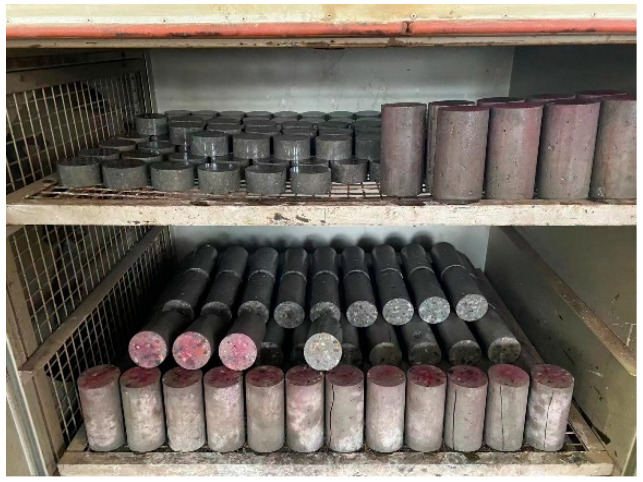
Specimen Drying.

A dry–wet cycle method according to “Standard for test methods for long-term performance and durability of ordinary concrete (GB/T 50082-2009)” [30] was performed. After the specimens were dried in an oven (Nantong Hunan Scientific Instrument Co., Ltd., Nantong, China) at (80 ± 5) °C for 48 h and cooled to room temperature naturally in a dry environment, the upper and lower surfaces of the specimens were sealed with wax. Then the specimens were placed in a soaking tank as shown in Figure 2. The distance between adjacent specimens was 20 mm, and the distance between the specimens and the side walls was not less than 20 mm. Prepared 5% Na_2_SO_4_ solution were injected into the soaking tank until it exceeded the upper surface of the specimens by 20 mm. A soaking duration of 12 h was applied. Upon completion of soaking, the specimens were retrieved and subjected to air-drying for 30 min. Following the air-drying step, the specimens were transferred to an oven and dried at a controlled temperature of (80 ± 5) °C for 6 h as shown in Figure 3. After the drying process was finished, the specimens were allowed to cool down to room temperature. Once cooled, the specimens were placed back into the soaking tank to commence the subsequent dry–wet cycle.

### 3.2. High Temperature

**Figure 4:**
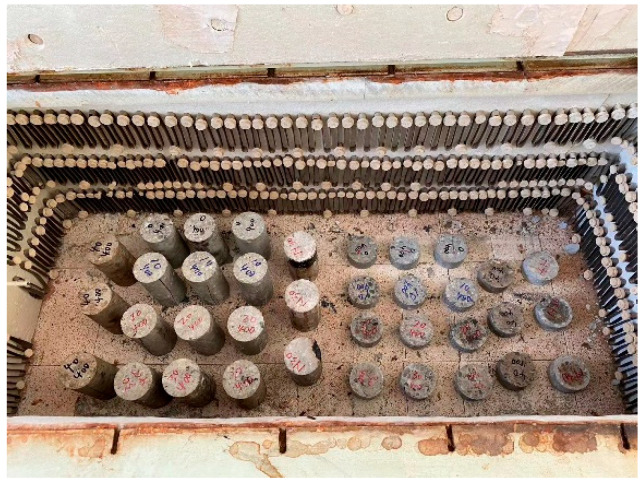
High-temperature test.

The high-temperature exposure was carried out in a high-temperature furnace (CGETEST Co., Ltd., Nanjing, China) in the Laboratory of Mechanical Properties of Civil Engineering Materials at Nanjing University of Aeronautics and Astronautics, as shown in Figure 4. The high-temperature furnace had a maximum target temperature of 1000 °C and a heating rate of 10 °C/min. After the sulfate corrosion, the wax present on the upper and lower surfaces of the specimens was removed. Before high-temperature exposure, the specimens were heated to 110 °C for 2 h to achieve complete drying. The specimens were cooled naturally to room temperature. The mass of the specimens was immediately measured following cooling. The cooled specimens were subsequently heated at a rate of 10 °C/min to the preset target temperature (200 °C, 400 °C, 600 °C, and 800 °C). The maximum target temperature of 800 °C was used as referenced in [12,13,14,15,16]. When the concrete reached 800 °C, the main component C-S-H (calcium silicate hydrate) had decomposed, and most compressive strength of the concrete had been lost [12]. Once the target temperature was attained, the specimens were held at this temperature for 3 h to ensure thermal equilibrium throughout the specimen. The specimens were cooled naturally to room temperature and their mass was weighed after cooldown.

**Figure 5:**
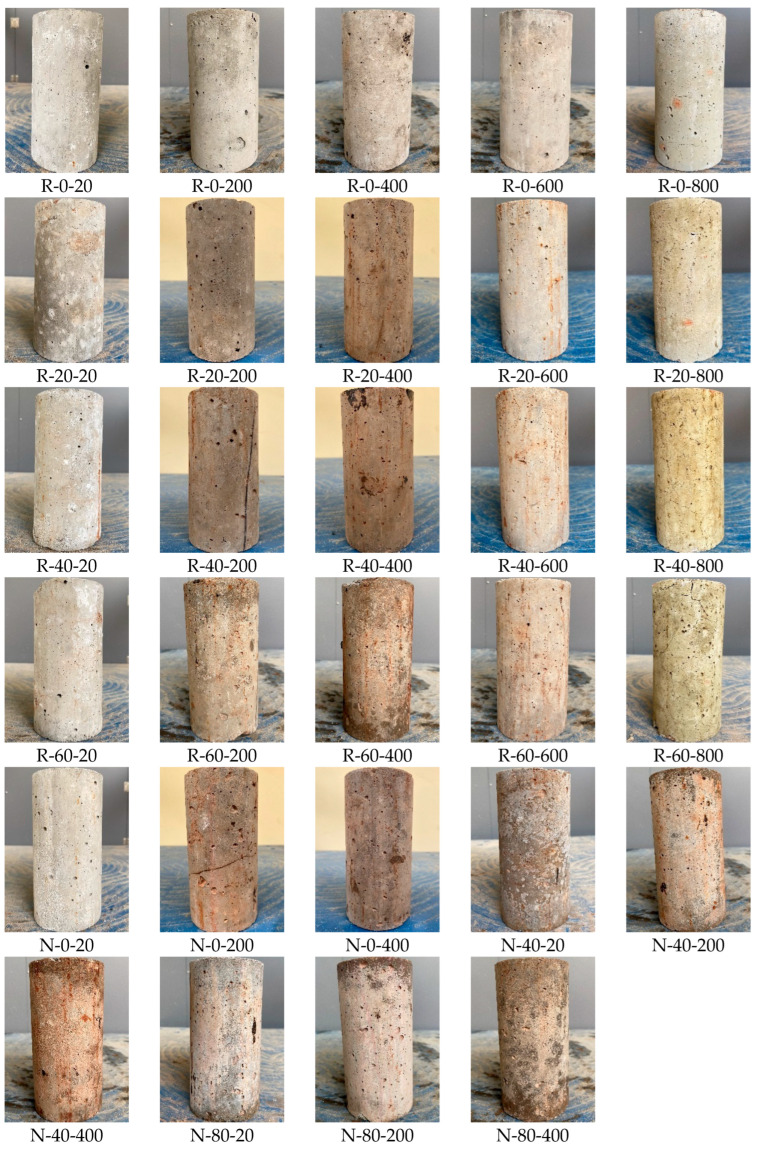
RAC and NAC specimens after different temperatures.

After high-temperature exposure, the surface of concrete specimens exhibited different colors at different temperatures as shown in Figure 5. The surface of specimen after 200 °C and 400 °C exposures turned slightly brown and a small number of small cracks were found. The color of the specimens at 600 °C and 800 °C turned yellow and white.

### 3.3. Quasi-Static Compression Test

**Figure 6:**
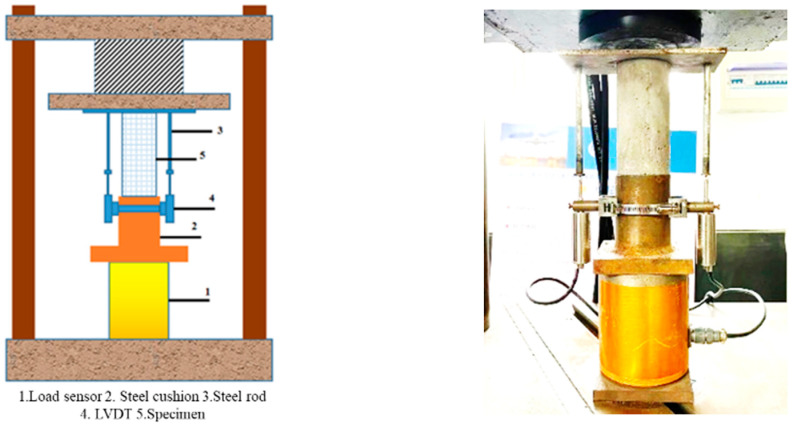
Test devices.

The quasi-static compression test was conducted on a hydraulic universal pressure tester YES-1000 (Jinnan Shijin testing mechine Co., LTD, Jinan, China), using a load rate of 1.0 kN/s (0.26 MPa/s) to control the loading speed. The axial load was measured using a 100t load sensor, and the longitudinal displacement of the concrete specimen was measured using LVDTs (Liyang Chaoyuan Instrument Factory, Liyang, China). Devices contained the load sensor and LVDTs, and a steel cushion and steel rods were applied to fix the specimen and the sensors, as shown in Figure 6. A dynamic acquisition instrument was used to collect the axial deformation and axial load of the relevant concrete specimen in real time during the quasi-static loading test process, and the acquisition instrument was the DHDAS dynamic acquisition system.

## 4. Test Results

### 4.1. Failure Mode

**Figure 7:**
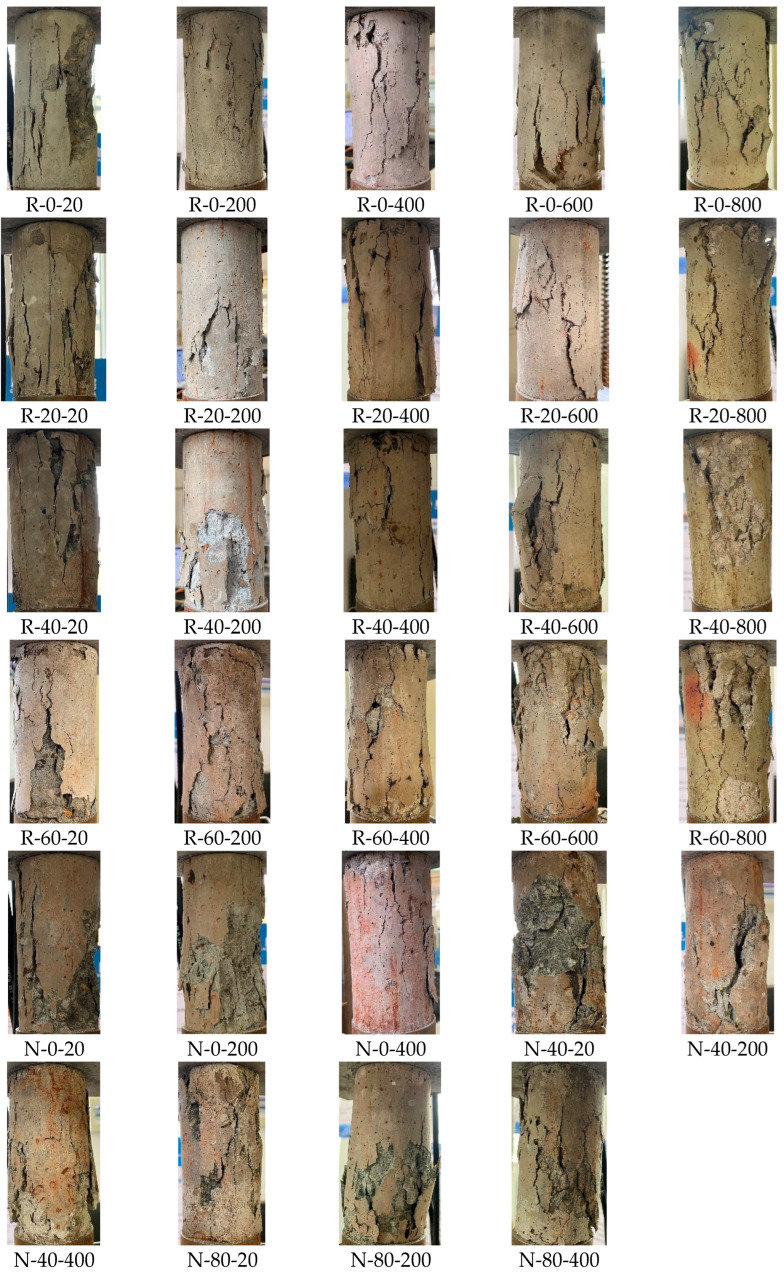
Failure Mode.

Figure 7 shows the compressive failure mode of concrete cylinder specimens after different high temperatures and different corrosion cycles. It can be seen that more spalling occurred with the increase in the number of dry and wet cycles, due to the initial damage caused by sulfate ions invading the interior of the concrete. After 20 °C and 200 °C temperature exposure, RAC and NAC specimens had typical brittle failure, showing a triangular cone-shaped cracking failure pattern. After the high temperature of 400 °C, the brittleness of the RAC specimen was greatly reduced. Cracks generated in the process of compression were connected with initial small transverse cracks appearing after high temperature, followed by the transverse expansion and the separation of aggregate and cement colloid. With the strengthening of the high-temperature softening effect, the transverse expansion was more serious. However, brittle failure mode could still be detected for NAC specimens, because the internal pores of NAC are less than that of RAC, and the internal changes after sulfate corrosion and high temperature were relatively lagging.

### 4.2. Stress–Strain Curves of RAC and NAC Under Uniaxial Compression

The compressive stress of the specimen is calculated according to Equation (1).

(1) $$\sigma =\frac{F_{v}}{S_{v}}=\frac{F_{v}}{\pi \times 35\times 35}\times 10^{3}$$

where σ is the axial compressive stress of the specimen (MPa), $F_{v}$ is the axial compressive load during quasi-static loading test (N), and $S_{v}$ is the cross-sectional area of the specimen (mm^2^).

**Figure 8:**
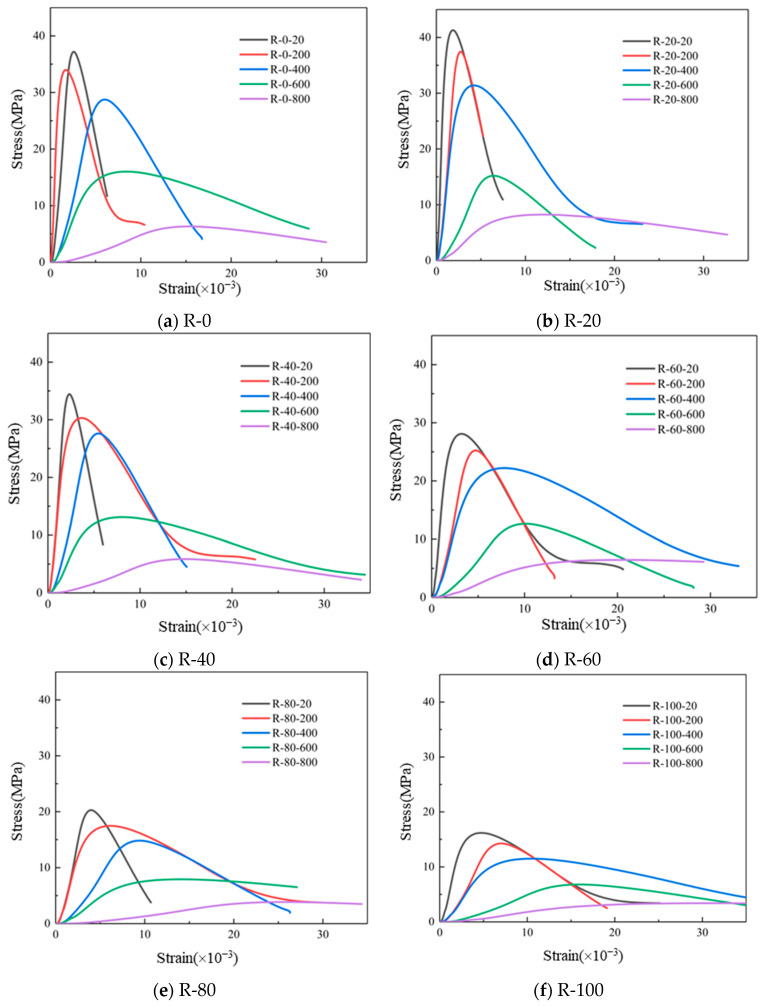
Stress–strain curves of RAC after different corrosion cycles and high temperatures for specimens of: (**a**) R-0; (**b**) R-20; (**c**) R-40; (**d**) R-60; (**e**) R-80; (**f**) R-100.

The compressive strain ε of the specimen is calculated according to Equation (2):

(2) $$\varepsilon =\frac{R_{v,1}+R_{v,2}}{2R_{v}}=\frac{R_{v,1}+R_{v,2}}{2\times 140}=\frac{R_{v,1}+R_{v,2}}{280}$$

where $R_{v,1}$ and $R_{v,2}$ are the measured longitudinal displacements (mm) and the length of the specimen (mm), respectively. The stress and strain values of concrete under each loading are determined by taking the average of the measured data from three specimens under the same condition. The uniaxial compression stress–strain curves are shown in Figure 8. The typical stress–strain curve was almost linear up to about 60% of the peak stress. When the load was increased to 80–100% of the peak stress, the slope of the stress-stain curve gradually decreased.

As the temperature increased, the trend of the RAC stress–strain curve undergoing different dry–wet cycles of sulfate corrosion gradually changed. At room temperature, the stress–strain curves of RAC showed linear increases in the ascending branch, which is consistent with the research results of Han et al. [31] on lightweight aggregate concrete. The descending portions of the stress–strain curves were steep, indicating that the specimens have obvious brittleness. With the increase in temperature, the peak stress of RAC specimens decreased and strain at peak stress (peak strain) of most RAC specimens increased. The slope of the ascending branch gradually decreased, resulting in a trend of flattening curve. After increasing dry–wet cycles of sulfate, the elastic modulus and peak stress of RAC decreased, while the corresponding peak strain gradually increased.

**Figure 9:**
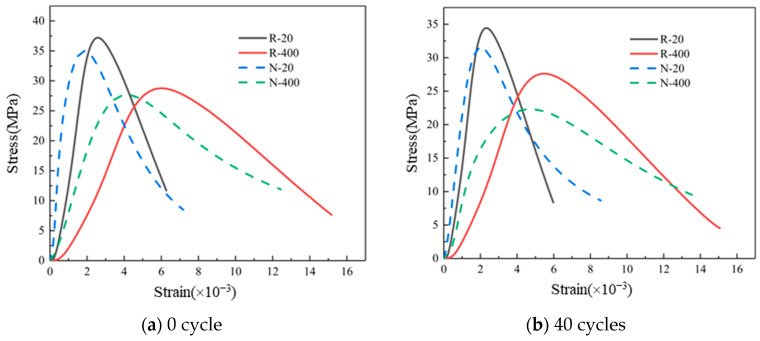
Stress–Strain curves of RAC and NAC: (**a**) 0 cycle; (**b**) 40 cycles of dry–wet sulfate corrosion.

The stress–strain curves of RAC and NAC that had undergone 0 and 40 dry–wet cycles of sulfate corrosion are shown in Figure 9. It can be seen that the stress–strain curves of RAC and NAC specimens at room temperature generally showed rapid rise, rapid decline after reaching the peak stress, and finally slow decline. The slope of the NAC curve in the ascending branch was higher, because the internal structure of NAC was more compact compared with RAC. At 400 °C, the peak strain increased and peak stress decreased when compared with that at room temperature. The increase in peak strain of the RAC was larger than that of NAC. This is because after the high temperature of 400 °C, the internal structures of RAC and NAC were damaged by the loss of structural water. There were more initial pores and microcracks in the RAC, further aggravating the damage after the high temperature. When the temperature was the same, with the increased dry–wet cycles of sulfate corrosion, the overall shape of RAC curve was slightly narrowed, while the shape of NAC curve was slightly slowed down. After 40 dry–wet cycles of sulfate corrosion, it is still in the process of filling internal defects of RAC with corrosion products, resulting in more compact internal structure. There were relatively few pores and micro cracks in NAC. After 40 dry–wet cycles of sulfate corrosion, the expansive corrosion products of NAC continued to fill the internal pores, resulting in internal damage and soft appearance.

### 4.3. Peak Stress

**Figure 10:**
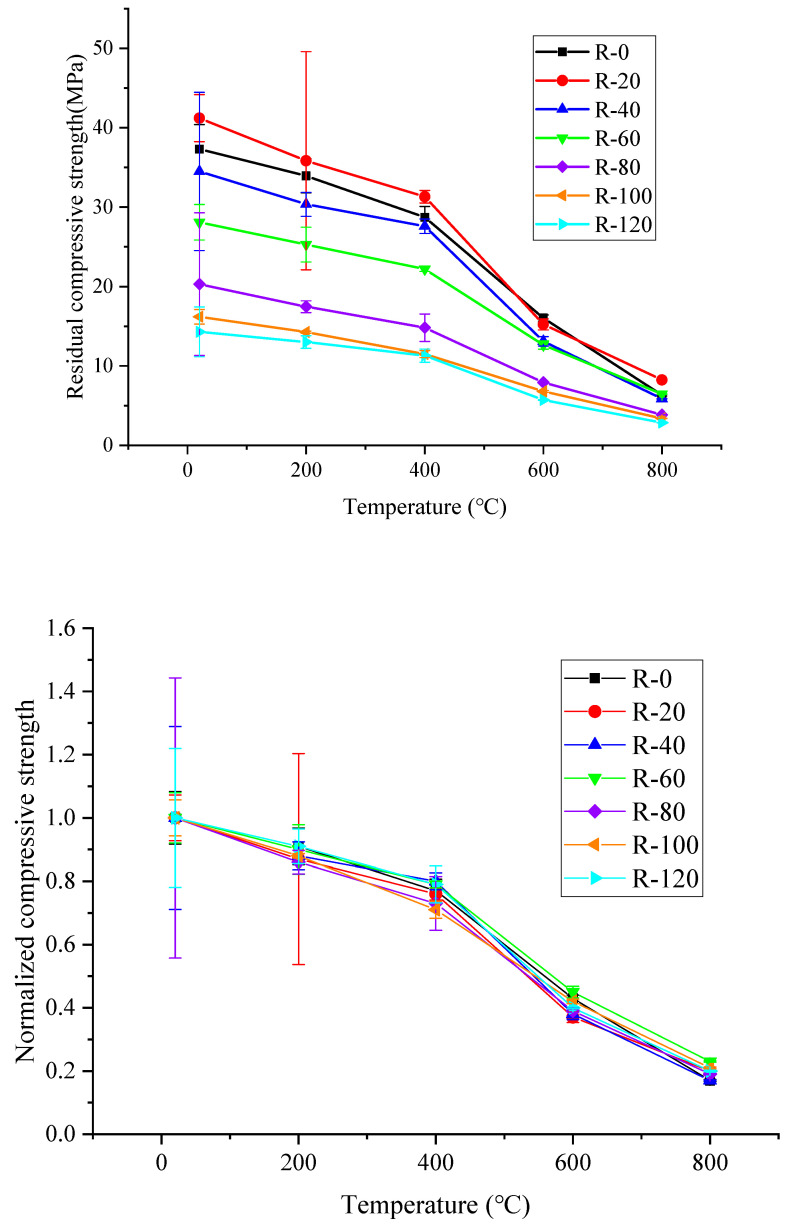
Strength and normalized strength of RAC after corrosion cycles and high temperatures.

The residual compressive strength after corrosion and temperature exposure (i.e., peak stress of stress–strain curves of RAC and NAC after corrosion and temperature exposure) and normalized residual compressive strength (residual compressive strength/residual compressive strength at room temperature) of RAC specimens after experiencing different high temperatures are shown in Figure 10. The peak stress of the RAC specimen at room temperature after 0, 20, 40, 60, 80, 100, and 120 dry–wet cycles of sulfate corrosion was 37.3 MPa, 41.2 MPa, 34.5 MPa, 28.1 MPa, 20.3 MPa, 16.2 MPa and 14.3 MPa, respectively. After the high temperature at 200 °C, the strength loss was small, and the normalized residual compressive strength was 0.91, 0.87, 0.88, 0.9, 0.86, 0.88 and 0.91, respectively. After the high temperature at 400 °C and 600 °C, the strength decreased. After the high temperature at 800 °C, the normalized residual compressive strengths were between 0.15 and 0.2.

After room temperature and 200 °C, the normalized compressive strength of RAC specimens decreased slowly with a decrease of 9%, 13%, 12%, 10%, 14%, 12% and 9% for 0, 20, 40, 60, 80, 100 and 120 dry–wet cycles of sulfate corrosion, respectively. Larger decrease was found after 400 °C, 600 °C and 800 °C. After the high temperature of 200 °C, the RAC specimens had slight damage due to the different temperature coefficients of mortar and coarse aggregate. Meanwhile, the bond between mortar and coarse aggregate was enhanced due to the dehydration and precipitation of bound water in mortar and coarse aggregate at 200 °C. Therefore, under the combined action of these two factors, the compressive strength of RAC specimens after high temperature of 200 °C is not varied much from that at room temperature. The deformation difference between mortar and coarse aggregate increased after high temperature, the initial crack continued to expand and extend, and the physical bond force between mortar and coarse aggregate decreased due to the damage of C-S-H gel dehydration after 400 °C exposures. After the high temperature of 600 °C, the crack width further expanded due to the expansion stress caused by the decomposition and crystallization of unhydrated cement particles. After the high temperature of 800 °C, the peak stress of the RAC specimen was almost lost, leaving only about 10% to 20% at room temperature.

**Figure 11:**
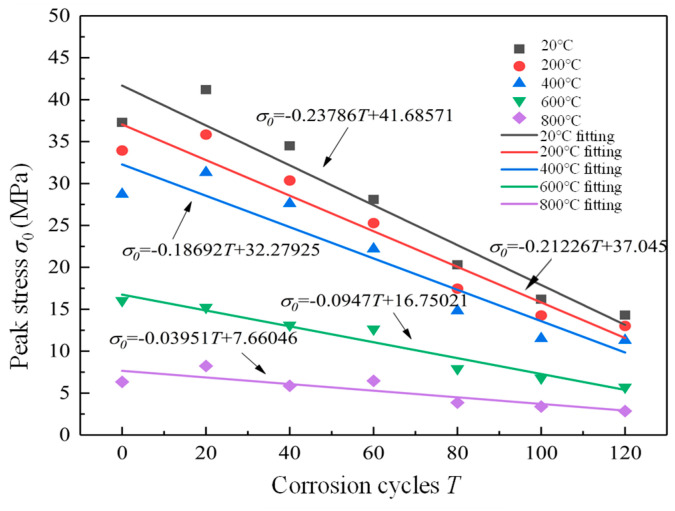
Peak stress versus corrosion cycles.

The relationship between peak stress and corrosion cycles is fitted as shown in Figure 11. It can be seen that the peak stress of the RAC specimen increased first and then decreased with the increase in corrosion cycles. The reason is that SO_4_^2−^ in salt solution reacts with calcium hydroxide (Ca(OH)_2_) and calcium aluminate hydrate (CaO·Al_2_O_3_·6H_2_O) in cement paste to form ettringite (3CaO·Al_2_O_3_·3CaSO_4_·32H_2_O) [19,20,21,22,23,24,25], and the solid volume increased by 94%. The early ettringite (3CaO·Al_2_O_3_·3CaSO_4_·32H_2_O) is filled in the pores of RAC, and the compactness increases, resulting in the increase in early compressive strength of RAC. In the later stage, due to the existence of three transition zones between the old and new interfaces in RAC, the permeability of RAC is greater than that of NAC due to this microstructure, which causes the SO_4_^2−^ in salt solution to penetrate into RAC more easily, and the continuously generated ettringite forms expansion stress in RAC. With the gradual increase in ettringite, the expansion stress increases, leading to accelerated formation and propagation of cracks in RAC and spalling of RAC. Therefore, the RAC strength decreased sharply in the late stage of corrosion. When the concentration of SO_4_^2−^ is high, not only ettringite (3CaO·Al_2_O_3_·3CaSO_4_·32H_2_O) will crystallize, but also gypsum (CaSO_4_·2H_2_O) will crystallize. The formation of gypsum will increase the volume of solid phase and also fill the pores of RAC at the early stage of erosion, leading to expansion and spalling of RAC at the later stage. The content of calcium hydroxide (Ca(OH)_2_) in the cement paste gradually decreases, and ettringite (3CaO·Al_2_O_3_·3CaSO_4_·32H_2_O) is produced at the same time, which will also cause the deterioration of RAC strength. The relevant linear fitting formula of RAC strength with corrosion cycle is shown in Equations (3)–(7).

(3) $$\sigma_{0}=-0.23786T+41.68571,R^{2}=0.953$$

(4) $$\sigma_{0}=-0.21226T+37.045,R^{2}=0.943$$

(5) $$\sigma_{0}=-0.18692T+32.27925,R^{2}=0.839$$

(6) $$\sigma_{0}=-0.0947T+16.75021,R^{2}=0.945$$

(7) $$\sigma_{0}=-0.03951T+7.66046,R^{2}=0.933$$

### 4.4. Peak Strain

**Figure 12:**
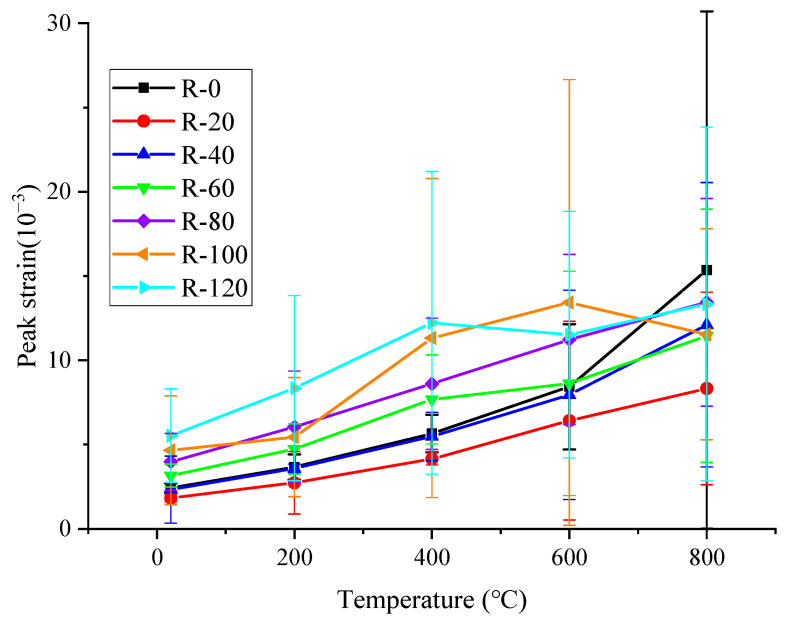
Peak strain of RAC after different corrosion cycles and high temperatures.

Peak strain is the strain corresponding to the peak stress. The variation in peak strain with temperature is shown in Figure 12. It can be seen that the most peak strain of RAC specimen gradually increases with the increase in temperature. When the temperature was less than 200 °C, the peak strain of RAC specimen increased slightly compared with that at room temperature. After high temperature at 200 °C, the peak strain of RAC specimens with 0, 20, 40, 60, 80, 100 and 120 dry–wet cycles of sulfate corrosion time increased by 51%, 49%, 54%, 50%, 52%, 51% and 51% compared with that at room temperature, respectively. In the temperature range from 200 °C to 800 °C, the increase amplitude gradually increased with the increase in temperature. After 400 °C, the peak strain of the RAC specimen increased rapidly with the increase in temperature, and the peak strain increased by 133%, 127%, 135%, 143%, 137%, 121%, 121% compared with that at room temperature, respectively. After 600 °C, the peak strain of RAC increased by 247%, 251%, 241%, 242%, 247%, 243%, 243%, respectively. After high temperature at 800 °C, the peak strain of RAC increased by 532%, 541%, 537%, 537%, 547%, 555% and 555%, respectively. It indicates that the specimen began to have obvious crack development in ITZ between mortar and coarse aggregate when the temperature was higher than 400 °C. By comparing the effects of different corrosion cycles and temperatures on the peak strain of RAC, it can be found that the corrosion cycle had a greater impact on the peak strain of RAC after temperature exposure lower than 400 °C, while the temperature had a greater impact on the peak strain of RAC in the temperature of 400–800 °C.

**Figure 13:**
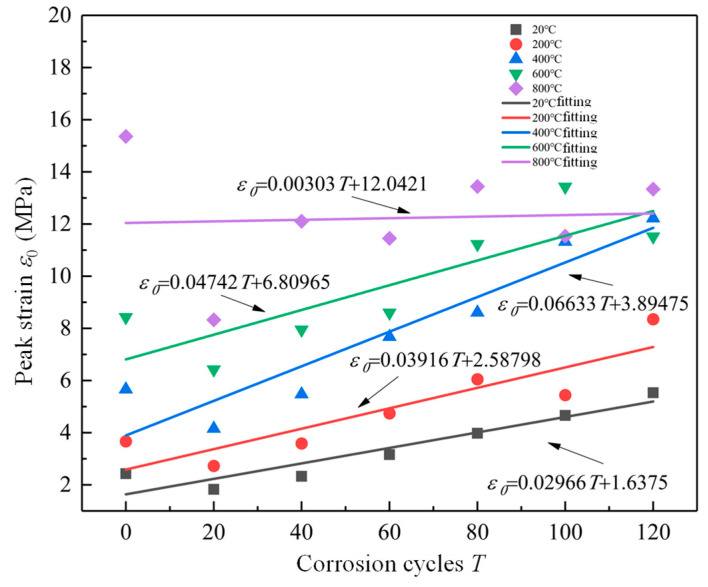
Linear fitting of peak strain with corrosion cycles.

The relationship between peak strain and corrosion cycles is fitted, and the fitting results are shown in Figure 13. It can be seen that the peak strain of RAC specimen increased almost linearly with the increase in corrosion cycles. SO_4_^2−^ successively entered the RAC, and the products generated by the reaction increased, resulting in damage to the RAC due to its inability to withstand the internal expansion pressure. The relevant linear fitting formula of peak strain with corrosion cycles is shown in Equations (8)–(12).

(8) $$\varepsilon_{0}=0.02966T+1.6375,R^{2}=0.936$$

(9) $$\varepsilon_{0}=0.03916T+2.58798,R^{2}=0.913$$

(10) $$\varepsilon_{0}=0.06633T+3.89475,R^{2}=0.893$$

(11) $$\varepsilon_{0}=0.04742T+6.80965,R^{2}=0.921$$

(12) $$\varepsilon_{0}=0.00303T+12.0421,R^{2}=0.935$$

### 4.5. Modulus of Elasticity

**Figure 14:**
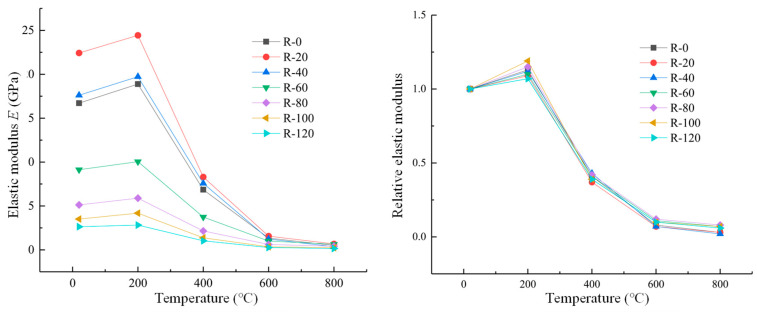
Elastic modulus and relative value of RAC after different corrosion cycles and high temperatures.

The elastic modulus $E_{c}$ of the concrete used in this paper is the secant modulus corresponding to 40% of the peak stress from the origin in the ascending section of the measured stress–strain curve. The elastic modulus ($E_{c}$) is calculated by the following expression [32,33]:

(13) $$E_{c}=\frac{\sigma_{0.4}}{\varepsilon_{0.4}}$$

where $\sigma_{0.4}$ is the stress corresponding to 40% of the peak stress in the ascending section, and $\varepsilon_{0.4}$ is the corresponding strain of $\sigma_{0.4}$. It should be noted that the elastic modulus is measured in a single loading cycle to compare the behavior of concrete, not to determine parameter values, which is usually measured by testing concrete in several cycles. Figure 14 shows the relationship between RAC elastic modulus and relative value of RAC after different corrosion cycles and high temperatures. It can be seen that the trend of elastic modulus with temperature is similar to that of peak stress. After temperature at 200 °C, the elastic modulus had a small increase compared with that at room temperature. When the temperature exceeds 200 °C, the elastic modulus began to decrease gradually with the increase in temperature, and the maximum decrease was about 60% after the high temperature of 400 °C. After temperature at 800 °C, the elastic modulus is only about 7% at room temperature. Due to the cracks in ITZ between mortar and coarse aggregate under high temperature, the decline in the elastic modulus of the RAC specimen is high, and the aggregate itself is also damaged to varying degrees after high temperature.

**Figure 15:**
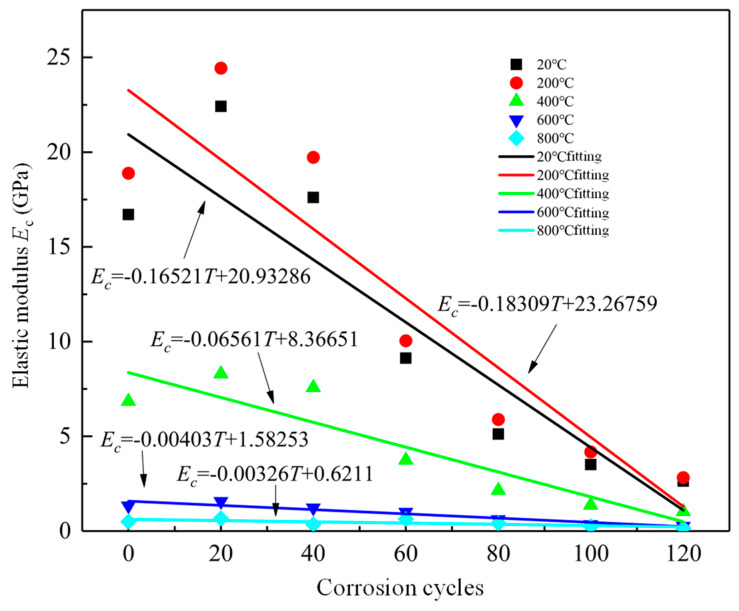
Fitting curves between elastic modulus and corrosion cycles.

Figure 15 shows the elastic modulus of RAC specimens with different corrosion cycles, and the relevant linear fitting formula of elastic modulus with corrosion cycles are shown in Equations (14)–(18). The variation in the elastic modulus is related to the peak stress and peak strain analyzed above.

(14) $$E_{c}=-0.16521T+20.93286,R^{2}=0.956$$

(15) $$E_{c}=-0.18309T+23.26759,R^{2}=0.912$$

(16) $$E_{c}=-0.06561T+8.36651,R^{2}=0.931$$

(17) $$E_{c}=-0.00403T+1.58253,R^{2}=0.961$$

(18) $$E_{c}=-0.00326T+0.6211,R^{2}=0.909$$

### 4.6. RAC Stress–Strain Curve Fitting

In this paper, the stress–strain curve model of uniaxial compression proposed by Guo [34] is used to fit the stress–strain curve of RAC specimens after sulfate corrosion under different high temperatures.

(19) $$y=\left\{\begin{array}{c} \frac{\sin\left(\frac{\pi }{2}ax\right)}{\sin\left(\frac{\pi }{2}a\right)},\;\;0\leq x<1\\ \frac{x}{b{\left(x-1\right)}^{2}+x},\;x\geq 1 \end{array}\right.$$

where $x=\varepsilon /\varepsilon_{0}$, ε is strain, $\varepsilon_{0}$ is peak strain, $y=\sigma /\sigma_{0}$, σ is stress, $\sigma_{0}$ is peak stress, *a* and *b* are the control parameters of the ascending section and descending section of the curve, respectively. The fitting results are shown in Table 5.

**Figure 16:**
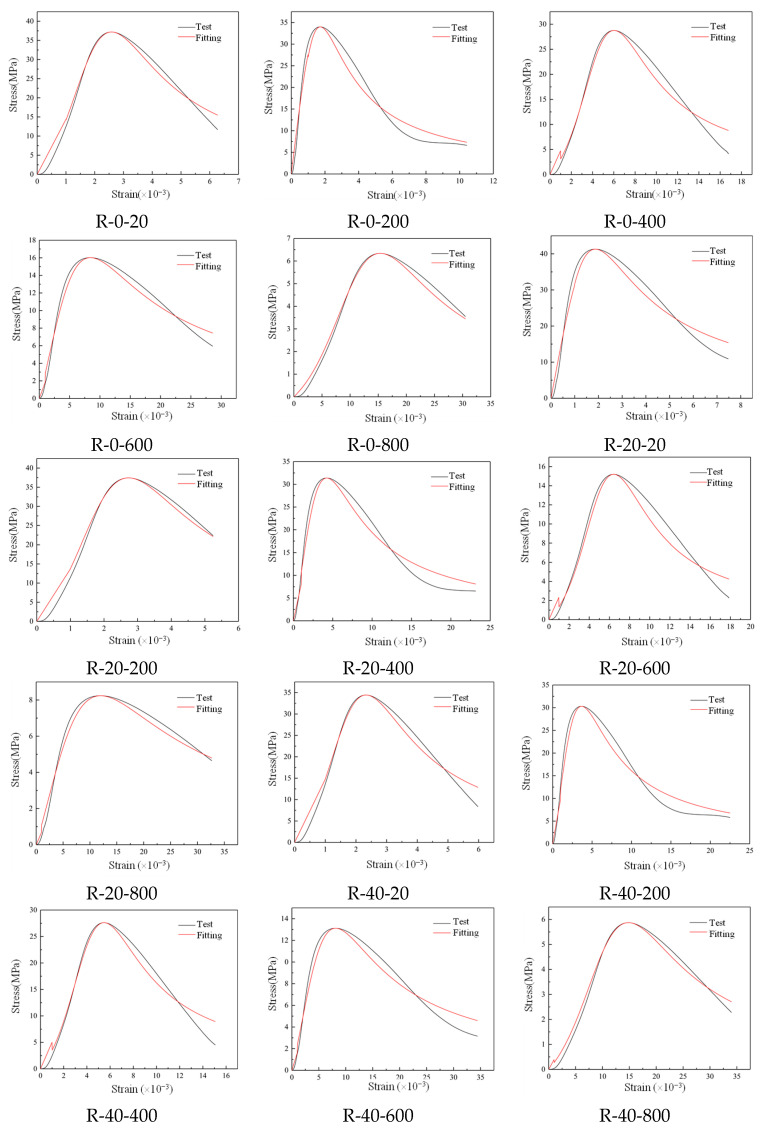
Test and fitting values of the RAC stress–strain curves.

Figure 16 shows the comparison of RAC test and fitting value. After 80 to 120 dry–wet cycles of sulfate corrosion, the RAC specimen was seriously damaged, and the shape of its stress–strain curve gradually tended to be flat. There was no obvious change in each stage, so the curve fitting in this paper is only for the specimens subjected to 0 to 40 dry–wet cycles of sulfate corrosion. As listed in Table 5, the values R^2^ of parameters *a* and *b* are mostly in the range of 0.950–1.000, which shows that the fitting curve is in good agreement with the test curve.

## 5. Conclusions

In this paper, the uniaxial compression test of RAC specimens after sulfate corrosion and high temperature was carried out. The conclusions obtained are as follows:

(1) After 200 °C high temperature and sulfate corrosion, the shape of RAC stress–strain curve was basically the same as that at room temperature; with the increase in temperature from 400 °C, the peak point of the RAC stress–strain curve had an obvious shift to the lower right, and the elastic stage of the ascending section of the curve was shortened, and the curve tended to be flat gradually; the increase in peak strain of the RAC was larger than that of NAC.

(2) After sulfate corrosion and high temperatures, the elastic modulus and peak stress of RAC decreased, while the corresponding peak strain increased gradually. When the temperature was lower than 400 °C, the number of dry–wet cycles of sulfate corrosion had a greater impact on the peak strain of RAC, while the temperature had a greater impact on the peak strain of RAC when the temperature was higher than 400 °C.

(3) Based on the experimental data, the uniaxial compressive stress–strain model proposed by Guo [34] was adopted to fit the stress–strain curves of the RAC subjected to 0–40 sulfate dry–wet cycles. The determination coefficients (R^2^) of the fitting parameters are mostly within the range of 0.950–1.000, indicating a high degree of agreement between the fitted curves and the experimental data. This model can effectively describe the compressive stress–strain behavior of RAC under the combined effects of sulfate corrosion and high temperature.

The tests were based on non-standard cylindrical samples (*Φ*70 mm × 150 mm) of RAC and NAC, and the applicability to samples with different shapes, sizes, or replacement ratios (e.g., 30% or 70%) remains unverified. Future work can focus on conducting field exposure tests or simulating complex practical environments to verify the applicability of current conclusions, testing concrete samples with varying aggregate replacement ratios, mix proportions, and geometric parameters to establish a more universal performance evaluation system, integrating macro-mechanical tests with microstructural analysis techniques (e.g., SEM, XRD) to reveal the intrinsic mechanism of strength degradation and build a multi-scale prediction model, and exploring the effects of admixtures (e.g., fly ash, silica fume) or surface treatment technologies to improve the corrosion and high-temperature resistance of RAC.

## Figures and Tables

**Table 1:** Physical and Mechanical Properties of Cement.

	Loss on Ignition (%)	Specific Surface Area (m^2^/kg)	Initial Setting Time (min)	Final Setting Time (min)	3d Strength (MPa)		28d Strength (MPa)	
					Flexural	Compressive	Flexural	Compressive
Measured	1.5 ± 0.1	332 ± 2	214 ± 1	258 ± 4	5.4 ± 0.2	27.4 ± 0.6	8.6 ± 0.3	51.2 ± 0.8
Required	≤5.0	≥300	≥45	≤600	≥3.5	≥17.0	≥6.5	≥42.5

**Table 2:** NCA and RCA properties.

Type	Bulk Density (kg/m^3^)	Apparent Density (kg/m^3^)	Crushing Index (%)	Water Absorption (%)	Dust Content (%)	Clay Lump (%)
NCA	1350 ± 107	2774 ± 76	13.2 ± 2.8	1.1 ± 0.1	0.30 ± 0.03	0.11 ± 0.08
RCA	1295 ± 6	2542 ± 71	14.2 ± 0.7	5.1 ± 0.8	2.5 ± 0.5	0.9 ± 0.3

**Table 3:** Concrete proportions.

Replacement of Recycled Aggregate	Water (kg/m^3^)	Cement (kg/m^3^)	Water-Cement Ratio	Sand (kg/m^3^)	NCA (kg/m^3^)	RCA (kg/m^3^)
0%	185	336.4	0.55	638.7	1239.9	0
100%	195	433.3	0.45	602.3	0	1169.4

**Table 4:** Specimens Design.

Specimens	Sulfate Corrosion		High-Temperature Test		Quasi-Static Test	
	φ70×140mm				φ70×140mm	
	Test Group	Total Specimen	Test Group	Total Specimens	Test Group	Total Specimens
R-0-20	1	3	1	3	1	3
R-0-200	1	3	1	3	1	3
R-0-400	1	3	1	3	1	3
R-0-600	1	3	1	3	1	3
R-0-800	1	3	1	3	1	3
R-20-20	1	3	1	3	1	3
R-20-200	1	3	1	3	1	3
R-20-400	1	3	1	3	1	3
R-20-600	1	3	1	3	1	3
R-20-800	1	3	1	3	1	3
R-40-20	1	3	1	3	1	3
R-40-200	1	3	1	3	1	3
R-40-400	1	3	1	3	1	3
R-40-600	1	3	1	3	1	3
R-40-800	1	3	1	3	1	3
R-60-20	1	3	1	3	1	3
R-60-200	1	3	1	3	1	3
R-60-400	1	3	1	3	1	3
R-60-600	1	3	1	3	1	3
R-60-800	1	3	1	3	1	3
R-80-20	1	3	1	3	1	3
R-80-200	1	3	1	3	1	3
R-80-400	1	3	1	3	1	3
R-80-600	1	3	1	3	1	3
R-80-800	1	3	1	3	1	3
R-100-20	1	3	1	3	1	3
R-100-200	1	3	1	3	1	3
R-100-400	1	3	1	3	1	3
R-100-600	1	3	1	3	1	3
R-100-800	1	3	1	3	1	3
R-120-20	1	3	1	3	1	3
R-120-200	1	3	1	3	1	3
R-120-400	1	3	1	3	1	3
R-120-600	1	3	1	3	1	3
R-120-800	1	3	1	3	1	3
N-0-20	1	3	1	3	1	3
N-0-200	1	3	1	3	1	3
N-0-400	1	3	1	3	1	3
N-40-20	1	3	1	3	1	3
N-40-200	1	3	1	3	1	3
N-40-400	1	3	1	3	1	3
N-80-20	1	3	1	3	1	3
N-80-200	1	3	1	3	1	3
N-80-400	1	3	1	3	1	3

**Table 5:** Fitting results.

Specimen	*a*	R^2^	*b*	R^2^
R-0-20	1.189	0.963	1.672	0.954
R-0-200	1.039	0.978	0.571	0.986
R-0-400	1.175	0.979	1.978	0.959
R-0-600	1.430	0.971	0.683	0.986
R-0-800	0.869	0.960	1.714	0.940
R-20-20	1.028	0.992	0.747	0.993
R-20-200	1.507	0.982	1.568	0.984
R-20-400	0.464	0.954	0.788	0.964
R-20-600	1.116	0.984	2.256	0.992
R-20-800	1.208	0.993	0.656	0.990
R-40-20	1.301	0.996	1.752	0.936
R-40-200	0.657	0.990	0.807	0.975
R-40-400	1.179	0.944	1.879	0.965
R-40-600	1.376	0.958	0.749	0.983
R-40-800	0.735	0.979	1.609	0.947

## Data Availability

The original contributions presented in this study are included in the article. Further inquiries can be directed to the corresponding author.
